# The mechanics clarifying counterclockwise rotation in most IVF eggs in mice

**DOI:** 10.1038/srep43456

**Published:** 2017-03-03

**Authors:** Kenta Ishimoto, Masahito Ikawa, Masaru Okabe

**Affiliations:** 1 The Hakubi Center for Advanced Research, Kyoto University, Kyoto, Japan; 2 Research Institute for Mathematical Sciences, Kyoto University, Kyoto, Japan; 3 Research Institute for Microbial Diseases, Osaka University, Suita, Japan

## Abstract

In mammalian fertilization, a small spermatozoon interacts with an egg that is a few thousand times larger in volume. In spite of the big difference in size and mass, when spermatozoa are bound to eggs, they begin rotating the eggs in *in vitro* observation. This was dubbed the ‘fertilization dance’. Interestingly, some papers reported that the rotation was counterclockwise, although the reason for this skewed rotation was not clarified. We focused on a chirality of helical beating of spermatozoa and found that eggs rotate counterclockwise in simulations under a certain geometrical condition where the eggs were situated. This theory of egg rotation was validated by demonstrating egg rotation in a clockwise direction by floating eggs to the upper surface of the IVF medium. The enigma of skewed rotation of IVF eggs was clarified.

Standard *in vitro* fertilization procedure involves introducing spermatozoa to eggs collected from the reproductive tract and incubating them under an appropriate condition. The fertilization process proceeds autonomously by introducing spermatozoa to eggs. The sperm-egg interaction starts with the binding of spermatozoa to the zona pellucida that surrounds each egg. The vigorously moving spermatozoa are captured on the surface of the zona upon contact by a strong affinity between the two. When several spermatozoa are bound to the zona pellucida, the eggs start to rotate by sperm flagella movement, sometimes referred to as a “fertilization dance”^1^. The rotation has been found to be directly proportional to the efficiency of *in vitro* penetration or fertilization competency of spermatozoa^2^,^3^. Interestingly, as these authors have already mentioned and many researchers have observed, most sperm-bound eggs rotate counterclockwise (when viewed from above). However, the reason for this preferential directionality has remained an enigma. In fact, a theoretical analysis indicated the directionality should be at random^1^.

In the present paper, the observed egg rotation was analyzed by numerical simulations using a mathematical model based on biomechanical theories. As a result, we could reason why the eggs rotate preferentially in a counterclockwise direction as described below.

## Results

### Observation of egg rotation in a dish

**Figure 1:**
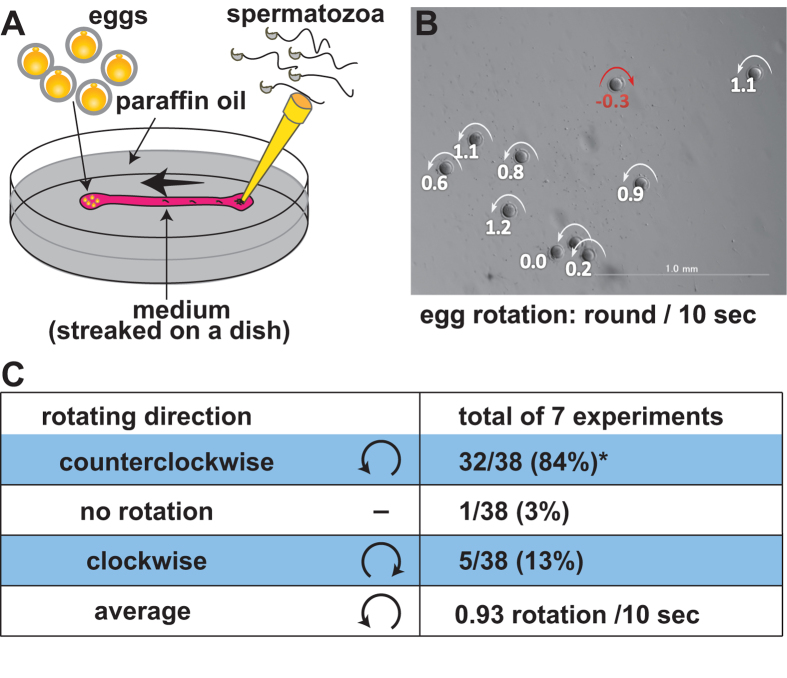
Observation of egg rotation in a dish. (**A**) An experimental setup to observe egg rotation. (**B**) A snapshot of the egg rotation from Supplemental Movie 1. Egg rotation in individual eggs was measured by the movie and expressed as round/10 seconds (the counterclockwise direction was defined as positive values.). (**C**) Observations of 38 eggs (total) were summarized in (**C**). ^*^The skewed rotating direction was proven statistically by the binomial test (p < 0.001).

In order to analyze the counterclockwise egg rotation, we placed eggs on one end of a streak of TYH (or FHM) medium^4^,^5^ and spermatozoa were added from the opposite end (Fig. 1A). The spermatozoa reached and bound to the eggs one by one. After 3 to 4 min, a few spermatozoa accumulated on the zona pellucida and the eggs started to rotate. As shown in Fig. 1B and in Supplemental Movie 1, the 10-second egg rotation was measured retrospectively. As a result, 32 of the 38 eggs observed rotated in a counterclockwise direction (Fig. 1C).

### Basics of sperm rotation

**Figure 2:**
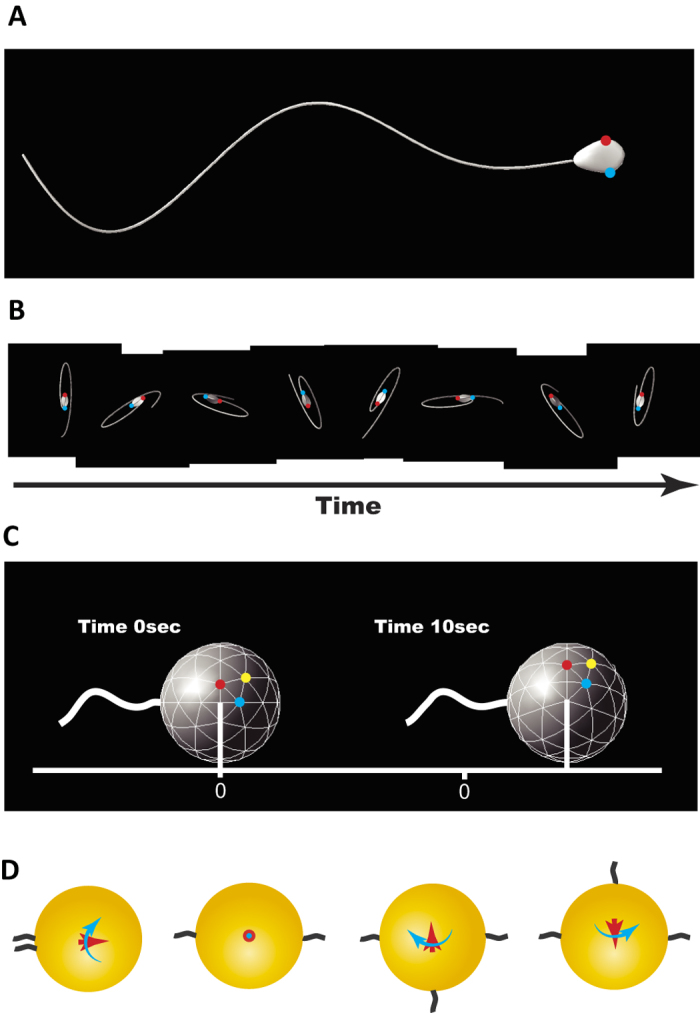
A simulation of sperm movement with helical waveform. (**A**) A snapshot of a model spermatozoon with helical waveform (Supplemental Movie 2). The red and blue spots indicate the edges of the widest part of the flattened head. (**B**) Time course of the simulated swimming spermatozoon with the same helical waveform as illustrated in A, viewed from the head side (Supplemental Movie 2). Each snapshot was taken at intervals of 0.4 beat cycle, and was vertically shifted so that the centers of the head are horizontally aligned. The red and blue spots correspond to the same points shown in A, demonstrating that the head rotates clockwise as time progresses. (**C**) Simulation of a single swimming spermatozoon connected to an egg. The translation and rotation after 10 seconds are shown, though the flagellum is emphatically illustrated for visualization. The spermatozoon exerts force and torque, and in turn, the egg is moved and rotated clockwise when viewed from the head. The egg rotation can be recognized by the marked points. The estimated rotation velocity by a single spermatozoon is 0.34 rpm, which might be insufficient for recognition in real observation. **(D**) Schematic pictures of the force and torque exerting on the egg (yellow) in sample cases with simple sperm configuration (black), viewed from above. The red arrows indicate the total force exerting on the egg, and the rotation direction by the whole torque is shown by blue arrows. When two spermatozoa attached from opposite directions, both the net force and torque vanish (second from the left), while the egg may rotate right or left by the binding of a third spermatozoon.

As experimentally observed^6^, mouse sperm head exhibited a clockwise rotation when viewed from the proximal end. The movement could be simulated by applying right-handed helical waveform^7^ to sperm tail which generates torque as well as propulsion for progression. In other words, the head rotates clockwise by a counter-rotation (relative to the head) of the tail to balance the total torque within the sperm cell. We computed hydrodynamic forces and swimming trajectory of model mouse spermatozoa using the boundary element method^8^, given the helical flagellar waveform as in our previous paper^9^. The computed swimming with clockwise head rotation is shown in Fig. 2A and B with snapshots and in Supplemental Movie 2.

We then computed a swimming spermatozoon attaching to the egg surface. The exertion by the spermatozoon enabled the egg to move slightly in one direction and rotate clockwise when seen from the proximal end, with small oscillation due to the flagellar beat (Fig. 2C). From the computed displacement of the egg, we estimated the force and the torque by a single spermatozoon as 5.41 pN and 2.28 × 10^2^ pNμm, respectively (see Methods).

**Figure 3:**
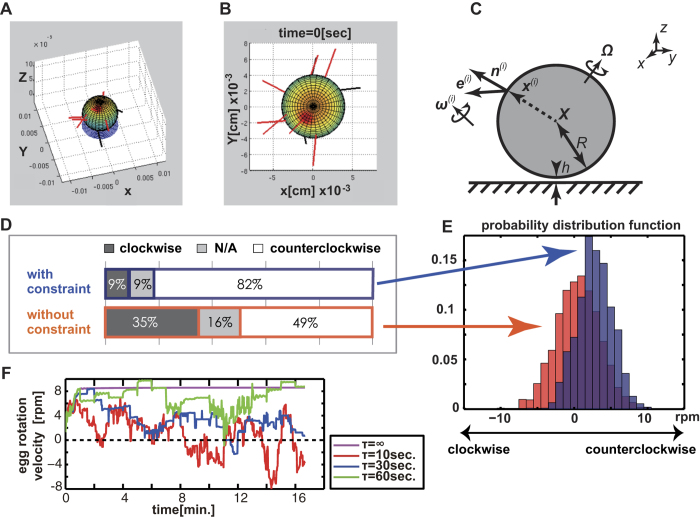
Simulation of the counterclockwise egg rotation. (**A**) A schematic illustration of our mathematical model of the egg rotation. The eggs were colored for presentation and are located near a bottom substrate, while the spermatozoa are depicted by red and black rods. The modeling is such that the spermatozoa in red are active, and generate forces and torques. In contrast, the black spermatozoa (with flagella intersecting the substrate, as shown in A and B) do not exert any force or torque on the egg (Simulation 2 to Simulation 4). (**B**) Top view of a snapshot of the typical movement of the egg simulated in the model fixed to the egg center. (**C**) The mathematical description of the model of a sperm-egg complex. Multiple rod-like spermatozoa were connected to a spherical egg located near a bottom substrate with a finite separation. The details of the symbols and the model are given in Methods. **(D**) The direction of the simulated egg rotation based on the model with and without the restriction on the spermatozoa that penetrated the substrates (Simulation 1 and 2), obtained by 2000 simulations with random initial sperm binding site. Each simulation was done with a randomized initial sperm binding site (Simulations 1 and 2). The figure illustrates the distribution of egg rotation velocity at *t* = 10 sec, as obtained in D. (F) Typical time evolution of rotation velocity of a sperm-egg complex with the sperm detach-attach process (Simulation 4). Note that the rotation direction may reverse occasionally when the sperm attachment sites are changed during the simulation process. Color indicates the simulation with a different detach-attach time period *τ* (Method).

As shown in Fig. 2D, the egg rotation and transition could be calculated as the sum of sperm exertion transferred to an egg. We randomized the sites to which the sperm attached, set the sperm numbers to 10, and analyzed whether or not the egg rotated. The varying number of spermatozoa from 10 to 30 did not affect the simulation outcome. The simulation results using sperm number 10 (as typically seen in real eggs) are shown in Fig. 3D.

### Simulation of the egg rotation

The sperm cells were postulated as straight rods with an effective length. To simplify our calculation, we assumed the force exertion from each spermatozoon was constant but the binding angle was flexible, while the spermatozoa were connected to the egg surface from random directions. Their exertion would yield egg motion, which in turn would give rise to hydrodynamic drag on spermatozoa moving with the egg. This would potentially change the binding angle in time, and such sperm-egg coupled dynamic was mathematically modeled (Methods, Fig. 3A to C).

In our model, a spherical egg was moved by 10 constant point forces and torques obtained by the detailed hydrodynamic simulation of a single spermatozoon attaching to the egg. The three-dimensional motion of the egg, including translation and rotation, obeyed the Stokes dynamics of zero net force and torques. The orientation of the 10 model rod-like spermatozoa evolved in time, following torque equations. The hydrodynamic torque by each model spermatozoon estimated by the resistive force theory^10^ was balanced by model elastic restoring torque that is linear to the angle between the initial sperm orientation and the instantaneous sperm orientation. The hydrodynamic interaction with the no-slip boundary was considered for the egg motion in the resistive matrices from the lubrication theory, whereby the sperm-boundary hydrodynamics interaction was neglected. See Methods for more details on the modeling.

Computer simulations on a total of 2000 virtual sperm-egg complexes (Simulation 1) revealed that the eggs rotated, but that the direction was not skewed counterclockwise as it was in the real eggs. The average rotational velocity after 2000 runs was 0.53 ± 2.95 rpm (mean ± SD, the direction of counterclockwise rotation was defined as positive) (Fig. 3D bottom and red histogram in 3E). As easily imagined, the net force and torque from variously binding spermatozoa cancelled each other. Thus, some manner of symmetry breaking should be included in Simulation 1 to simulate skewed rotation.

### Emergence of a counterclockwise rotation

In Simulation 2, we conditionally cancelled the movement of spermatozoa when any sperm tail crossed the virtual plane on which eggs were placed, considering the geometrical hindrance of sperm movement by a glass substrate. In 2000 runs of simulation, we found the rotation skewed to a counterclockwise direction with an average rotational velocity of 2.80 ± 2.39 rpm. (Fig. 3D top and blue histogram in 3E). A typical simulated movement of the egg was shown in Supplemental Movie 3. An asymmetric distribution of spermatozoa on eggs stemming from the existence of a bottom surface was the cause of the skewed direction of egg rotation. However, about 15% of the time, the sperm distribution favored the clockwise rotation in randomized simulation and also in real eggs. Thus, a symmetry break of the configuration geometry was indicated to play an important role in determining the direction of rotation.

We initially thought the change in beating direction of sperm tails might contribute to accelerating egg rotation (positive feedback effect). However, when the binding angles were kept at random, the rotating velocity only decreased by about 10% (2.59 ± 2.19 rpm) in simulation (Simulation 3).

Although we found the conditions to reproduce a counterclockwise rotation in simulation, the direction of the rotation in each run was fixed (clockwise or counterclockwise) different from that of real eggs.

### Occasional reversal of rotation

The real sperm-zona binding is a reversible phenomenon. Some spermatozoa detached during the observation of the egg rotation. In fact, one report demonstrated that numerous spermatozoa bound to the zona were replaced by a new group of spermatozoa within an hour^11^. By introducing a sperm replacement step in the simulation (Simulation 4, see Methods), the counterclockwise rotation remained dominant, but a reverse in rotation direction appeared occasionally (Fig. 3F, Supplemental Movie 4) as in the real eggs.

### Sliding motion

When the number of spermatozoa on the zona increased, the exertion from the spermatozoa caused real eggs to float up from the bottom of the dish, often yielding a seemingly sliding motion. This was successfully reproduced in Simulation 5 (Supplemental Movie 5). It should be noted that the frictional force^12^ pinning the egg and preventing it from sliding along the bottom plays an important role in forming a spinning motion. Once the frictional force was decreased as the eggs floated up, the eggs were easily moved in one direction. When the real eggs slid, the spermatozoa were observed spontaneously changing to a leeward direction for binding, which functioned to rotate eggs cooperatively. This rotation to a sliding transition was also reproduced in our model.

### Artificially produced clockwise rotation in real eggs

**Figure 4:**
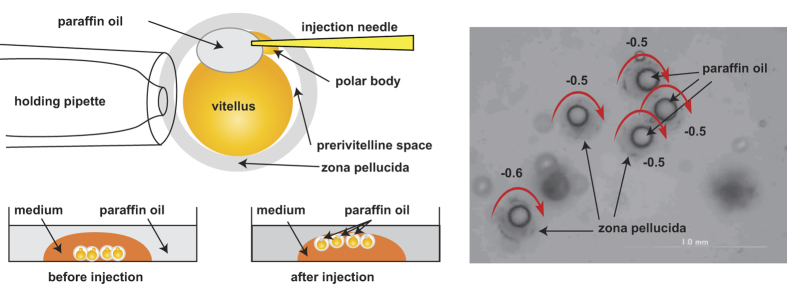
Artificially reversed egg rotation. (**A**) A schematic diagram of the oil injection to float the eggs. The eggs were floated near an oil-water interface, where the no-slip boundary conditions could be applicable due to the high viscosity of oil. (**B**) A snapshot from the Supplemental Movie. Observation, together with the egg rotation speed, taken as positive for counterclockwise rotation when viewed from the top (Supplemental Movie 6). The average rotation was −3.12 rpm (clockwise rotation).

Our simulation indicated that the symmetry break brought by the bottom of the dish is the major cause of counterclockwise rotation. For experimental verification, we injected paraffin oil into the perivitelline space of the eggs. This caused eggs to float up to the surface of the medium, with the symmetry of sperm-egg binding broken by the sealing instead of the bottom floor. As predicted by the simulation, the eggs were shown to rotate in a clockwise direction (Fig. 4 and Supplemental Movie 6).

## Discussion

There were at least two preceding examples of literature describing the egg rotation phenomenon in which the zona-free hamster eggs rotated counterclockwise by hamster^3^ and by human spermatozoa^2^,^3^. Furthermore, a renowned scientific TV program recording the process of human fertilization by Lennart Nilsson indicated that the human eggs surrounded by cumulus matrix were rotated by spermatozoa in a counterclockwise direction^13^.

As shown in the present paper, using mice as experimental models, we postulated that the counterclockwise rotation is caused by the right-handed helical waveform movement of sperm tail along with the geometrical hindrance by the presence of the bottom surface. We presume the counterclockwise rotation described in the precedent papers was also induced by the same mechanism which we saw in the mouse model.

However, there is no consensus concerning the direction of sperm tail movement in various species, probably due to the flexibility of swimming patterns of spermatozoa and different methods of observation in individual experiments. Ishijima *et al*. reported that human spermatozoa beat their tails in both directions^14^, and Kantsler *et al*. reported that human sperm tail movement changed depending on the environmental conditions of spermatozoa^15^.

Since the validity of our simulation model was certified by switching the rotating direction of the real eggs, we presumed that the counterclockwise rotation described in precedent papers using human and hamster spermatozoa indicated a right-handed direction of human and hamster sperm head movement in conditions under which they observed the egg rotations. It would be interesting if we could observe the egg rotation in species where the waveforms of spermatozoa are definitely clarified in the opposite direction for the complete validation of the present simulation.

Nilsson’s TV program^13^ includes a movie of rotating egg and the phrase “the egg rotation in the beginning of fertilization”. However, the fertilizing eggs are surrounded by massive cumulus cells and are placed in a narrow reproductive tract. Moreover, only a small number of spermatozoa are required to fertilize eggs *in vivo*^16^. The enigma of egg rotation *in vitro* has been clarified, though i*n vivo* eggs are likely fertilized without rotation.

## Methods

### Animals

All experiments reported here were approved by the Animal Care and Use Committee of the Research Institute for Microbial Diseases, in accordance with the Guidelines for Proper Conduct of Animal Experiments published by the Science Council of Japan. The ICR male and female mice were purchased from Japan SLC, Inc. (Shizuoka, Japan). Female ICR mice were superovulated by intraperitoneal injections of 5 U of equine chorionic gonadotropin (eCG) followed by injection of 5 U of human chorionic gonadotropin (hCG) 48 h later. Eggs were recovered 16 h after HCG injection and placed in a modified Krebs–Ringer bicarbonate solution (TYH medium) containing glucose, sodium pyruvate, bovine serum albumin, and antibiotics^4^. Eggs were treated with hyaluronidase (Sigma Type I-S, 150 units/ml) to remove cumulus layers and were kept in TYH medium until use.

The spermatozoa were collected from cauda epididymis of mature ICR mice and suspended in 100 μl TYH medium and were used for the experiments 1 hr to 2 hr after preparation, in a suspension containing up to 25% of acrosome-reacted spermatozoa^17^. The sperm tail motion is considered to change into hyperactivation around the time when spermatozoa accomplish capacitation, which is also the time when the acrosome reaction takes place^16^. In the present manuscript, we were not able to examine if the spermatozoa show hyperactivation. However, it was indicated that zona-binding spermatozoa are mostly acrosome intact replacing the acrosome reacted spermatozoa^11^. Combining these facts together, we imagined that the majority of swimming patterns of spermatozoa on zonae pellucidae to rotate eggs were not hyperactivated. The injection of oil into the perivitelline space of eggs was done using an Olympus IX-70 microscope equipped with a Narishige micromanipulator. The egg rotation was recorded by an Olympus DP30BW CCD camera using DP controller 3.2.1.276.

### Force estimation by simulation with a single spermatozoon

Both an egg and a spermatozoon are such small objects that all inertial effects are negligible or the Reynolds number is small enough so that dynamics can be completely determined by the time course of the deformation of the objects immersed in the medium^18^. Here, both the force exerted by the flagellum on the surrounding medium and the sperm swimming velocity were obtained, given the flagellar waveform of a mouse spermatozoon from the observational data. The computational approach based on hydrodynamics has recently succeeded not only in explaining experiments, but also in predicting physical quantities such as force by swimming spermatozoa which are difficult to assess by laboratory experiments^19^.

Woolley reported that mouse spermatozoa possess “twisted planar” flagellar waveform, which yields the clockwise head rotation as viewed from the sperm head^6^. However, the reported complicated head trajectory was later explained by the nearly planar but three-dimensional helical waveform by the hydrodynamic analysis^7^. We therefore have adopted this right-handed helical waveform as in their study^7^ and have implemented the direct numerical simulation scheme based on the boundary element method^8^,^20^, applying the ellipsoidal right-handed helical waveform.

In Fig. 2A,B, views of the computed swimming spermatozoon in medium are shown from the side and from the head, and the clockwise head rotation is well reproduced (see also Supplemental Movie 2).

As the sperm head is rather small compared with the egg size, we neglected the head part for the estimation of the force and the torque by a single spermatozoon upon the egg surface. Supposing the sperm flagellum is directly connected to the egg surface, the dynamics can be simulated by the same numerical scheme (Fig. 2C). We numerically computed the dynamics of the beating flagellum perpendicularly connected to the surface of the spherical egg. From the computed time-averaged translation and rotation of an egg, the force and torque exerted by the single spermatozoon can be obtained using Stokes’s law of drag. The force and torque by sperm swimming on the zona have been estimated in various species and different settings^21^,^22^,^23^. Our computational method also follows these literatures, but is based on a more accurate numerical scheme.

### Mechanical model of the egg rotation

Suppose a spermatozoon is a straight rod with length *L*′, and *N* sperm rods are connected to the surface of a spherical egg of radius *R*, being located near a bottom substrate (Fig. 3A–C). The physical parameters are provided in Supplemental Table 1. The parameters, *N* and *L*′, cannot be determined precisely for lack of information; in this model, they are therefore provided so that the values have orders of magnitude consistent with observation. It should be noted that changes in these values did not alter the main results qualitatively. Let the substrate be our *xy* plane, and vertical axis towards the medium be our *z* axis. The position and direction of each cell are denoted by **X** + ***x***^(*i*)^ and an unit vector ***e***^(*i*)^ (*i* = 1, …, *N*), respectively, where ***X*** = (*X, Y, Z*) is the center of the spherical egg. The orientation vector is taken towards the sperm tail end. The initial positions and contact angles of the spermatozoa appeared practically random from observations as shown in Supplemental Figure 2. The force and torques exerted by spermatozoa to the egg are given by 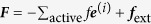, and 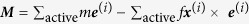, where the summation is taken over the cells which generate exertion, and in Simulation 1 and Simulation 5 all the cells are supposed to be active. But in the other simulation cases (Simulation 2 to Simulation 4), we neglected the exertion by spermatozoa when their tails crossed the *xy* plane. This constraint was introduced to take a geometrical hindrance by the presence of the substrate into consideration. The external force ***f***_ext_ is the summation of buoyancy and the repulsive surface potential force^9^. As the egg is slightly heavier than surrounding medium, this surface force guarantees an equilibrium distance between the egg and the substrate, *h*.

The egg dynamics follow to low Reynolds number physics, where the force ***F*** and the torque ***M*** are linearly dependent on the linear and rotational velocity, ***U*** and **Ω**, i.e. ***F*** = ***K***_*T*_***U*** + ***K***_***T***_***Ω***, 
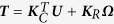
. Here ***K***_***T***_, ***K***_***C***_, and ***K***_***R***_ are 3 × 3 matrices called resistive matrices, only depending on the geometrical configuration, and the superscript *T* denotes the transpose of a matrix. In the presence of an infinite plane wall, the lubrication theory provides the asymptotic expression of the matrices as functions of (*Z* − *R*)/*R* (Appendix of Lauga *et al*.^12^, for the explicit values).

The dynamics of the attaching spermatozoa are obtained from the time evolution of the orientation vector, ***e***^(*i*)^, which is followed by the torque balance equation of each rod-like spermatozoon. Assume that the elastic moment at the point of attachment depends linearly on the angle between the initial sperm orientation 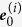 and the instantaneous sperm orientation ***e***^(*i*)^, and the angle is given by 
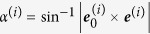
. The hydrodynamic resistive force is also linearly dependent on the local velocity. As the small hydrodynamic interaction between the cells is negligible, the torque balance equation may be written as 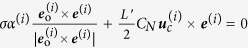, where the constant *σ* is the parameter corresponding to the effective bending stiffness, and 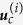 is the velocity of the center of the rod,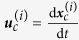 with 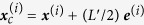. We estimated the order of the bending stiffness, following the value of human spermatozoa^19^. As the flagellum is sufficiently thin, the resistive force theory can be applied. Let *C*_*N*_ be the resistive coefficient with respect to the normal velocity, and we may write *C*_*N*_ = 4*πμ* (log(2*L*′/*a*) + 0.5)^−1^, where *μ* is the viscosity of the medium and *a* is the radius of the flagellum^11^. Here, the tangential component of the hydrodynamic drag on the spermatozoon is dropped, as it is negligible compared with the drag on the egg.

### Implementation of the computational simulation

For the time evolution, the 4th order Runge-Kutta method was used and the whole simulation is coded in Matlab*^®^*. To simplify the numerics, we introduced the body-fixed coordinates, 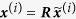 and 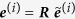, where ***R*** is the three-dimensional rotational matrix. After we defined the corresponding rotational velocity in the body-fixed frame, ***ω***^(*i*)^, as 
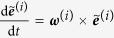
, we could derive the torque balance equation explicitly with respect to 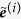. Note the gauge-fixing condition, 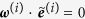, was used for the derivation.

The egg was initially located at 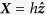, where 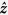 is the unit vector directed to the positive *z* axis. The spermatozoa were uniformly randomly attached on the surface, with contact angles that obey the cosine distribution. The physical and simulation parameters of the mouse spermatozoon and the egg are given in the Supplemental Table 1. The increase of the number of the attaching spermatozoa did not notably change the percentage of the rotation direction, though the rotational velocity was increased. The surface repulsion was induced so that the egg stayed at an equilibrium state around the initial separation *h* in the absence of the sperm exertion.

The judgment of the rotation direction, shown in Fig. 3D, was followed by the *z* component of the rotational velocity at *t* = 10 sec, 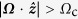, where we employed the critical rotational velocity, *Ω*_c_/2*π* = 0.1 sec^−1^, which corresponded to our experimental criterion.

#### Simulation 1

Sperm-egg complex model without constraint on spermatozoa that penetrated the substrate (Fig. 3D bottom and red histogram in Fig. 3E).

#### Simulation 2

Sperm-egg complex model with constraint on spermatozoa that penetrated the substrate (Fig. 3D top and blue histogram in Fig. 3E, Supplemental Movie 3). Only the constraint was incorporated in Simulation 1.

#### Simulation 3

Sperm-egg complex model without binding angle modulation in time. The effective bending stiffness *σ* was set as a very large value (*σ* = 1) from Simulation 2, in order to keep the bending angle in time.

#### Simulation 4

Sperm-egg complex model with random replacement of the attaching cells (Fig. 3F, Supplemental Movie 4). Occasional sperm-zona attachment and detachment were incorporated into Simulation 2. One of the spermatozoa that attach to the egg is randomly detached in every constant time period, *τ*, and, at the same time, a single spermatozoon is attached in a random place on the egg surface with a new random contact angle.

#### Simulation 5

Sperm-egg complex model without boundary effects (Supplemental Movie 5). The surface repulsive force was removed from Simulation 1, and the resistive matrix of the egg was additionally changed to that of an isolated sphere.

### Ethical Approval

All experiments reported here were approved by the Animal Care and Use Committee of the Research Institute for Microbial Diseases, in accordance with the Guidelines for Proper Conduct of Animal Experiments, Science Council of Japan.

## Additional Information

**How to cite this article**: Ishimoto, K. *et al*. The mechanics clarifying counterclockwise rotation in most IVF eggs in mice. *Sci. Rep.* **7**, 43456; doi: 10.1038/srep43456 (2017).

**Publisher's note:** Springer Nature remains neutral with regard to jurisdictional claims in published maps and institutional affiliations.

## Supplementary Material

Supplementary Materials

Supplementary Movie 1

Supplementary Movie 2

Supplementary Movie 3

Supplementary Movie 4

Supplementary Movie 5

Supplementary Movie 6
