# Supplementary material for: The mechanics clarifying counterclockwise rotation in most IVF eggs in mice
Source: Sci Rep. 2017 Mar 3;7:43456. doi: 10.1038/srep43456 (PMC5335560; doi:10.1038/srep43456)
Supplement: Supplementary Materials [file srep43456-s1.pdf]

Title: The mechanics clarifying counterclockwise rotation in most IVF eggs in mice

Supplementary Materials

Authors: Kenta Ishimoto[1,2*], Masahito Ikawa[3], Masaru Okabe [3*]

Affiliations:

[1] The Hakubi Center for Advanced Research, Kyoto University, Kyoto, Japan.

[2] Research Institute for Mathematical Sciences, Kyoto University, Kyoto, Japan

[3] Research Institute for Microbial Diseases, Osaka University, Suita, Japan

*Correspondence to: Kenta Ishimoto (ishimoto@kurims.kyoto-u.ac.jp)

Masaru Okabe (okabe@biken.osaka-u.ac.jp).

	meaning	value
R	egg radius	40 µm (23)
L	sperm flagellar length	80µm (22)
r	flagellar radius	0.25µm (16)
c	density offset of egg	0.02 *
T^{-1}	beat frequency	22Hz (18)

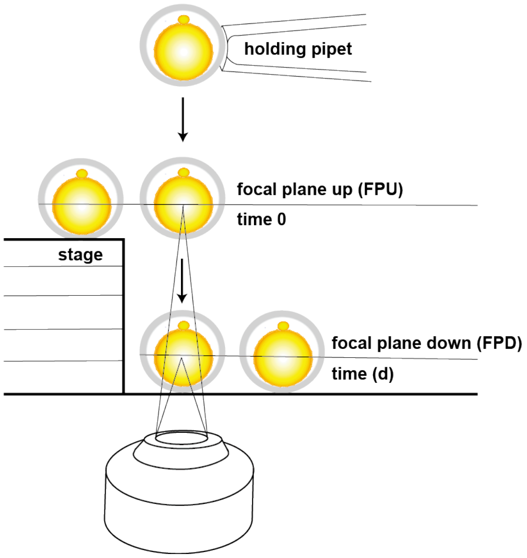

Supplemental Figure 1. A set of model parameters used in the numerical simulation.

* The density offset of egg was experimentally estimated. Briefly, an egg held by a manipulating pipet was dropped in the medium and the time required to sediment from the focus plane of the eggs placed on the 0.82 mm stage (made by 5 layers of 0.17mm cover glass)(FPU) to that of eggs placed in the bottom of the dish (FPD) was measured (12.3 ± 1.6 sec: mean ± s.d.). The calculation was done following Stokes' law.

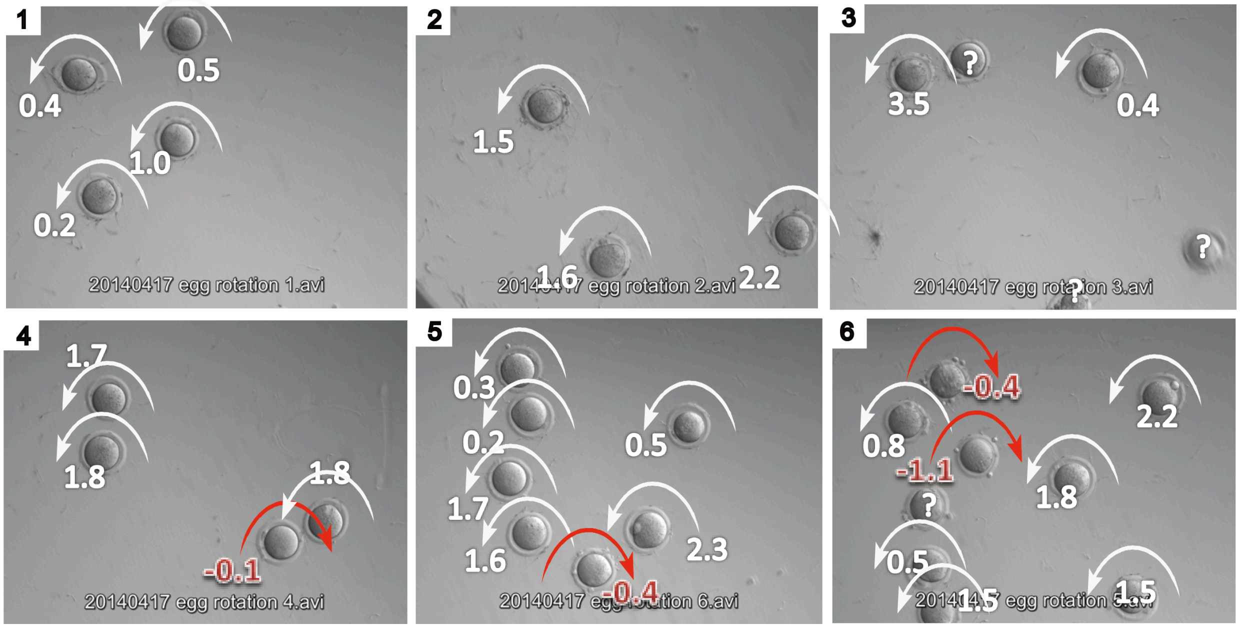

Supplemental Figure 2. Snapshots from Supplemental Movie 1.

The number of egg rotations is indicated beside the eggs. Counterclockwise rotation, when viewed from the top, is expressed as a positive value.

Supplemental Movie 1

The eggs were inseminated with spermatozoa as indicated in Figure 1. The eggs were photographed and the number of rotations was measured after defining the counterclockwise direction as a positive value. The figures were indicated as round /10 sec. (See also supplemental figure 2.)

Supplemental Movie 2

A model helical waveform swimming pattern of a spermatozoon was simulated based on the observation by Woolley [6]. The head was modeled as a deformed flattened ellipsoid, but the hook structure of the head was not incorporated. The simulation shows the head rotates clockwise as seen from the head, and the complicated cell rotation during swimming is sufficiently reproduced. The white plate under the cell indicates the fixed bottom substrate, to visualize the cell position in the reference frame, and the white meshes covering the sperm indicate the numerical meshes used in the boundary element method.

Supplemental Movie 3

Typical movement of the rotating egg computed based on the mathematical model with parameters given in the tables of Supplemental Fig. 1 (Simulation 2). The egg rotated counterclockwise in most simulation runs, when seen from above. The three-dimensional view of the egg and rod-like sperm cells is shown to the left. The active spermatozoa were depicted in red and inactive cells in black. The image to the right shows the sperm-egg cluster fixed to the egg center seen from above.

Supplemental Movie 4

Typical movement of the rotating egg simulated based on the mathematical model with the detachment and attachment process (Simulation 4). The parameters for simulation were used as in Supplemental Fig. 1, and the time period of the sperm mixing was $\tau = 10\text{sec}$. The egg could reverse the rotation direction by a random change in the position of sperm. The views and colors are as given in Supplemental Movie 3.

Supplemental Movie 5

Typical movement of the rotating egg simulated based on the mathematical model without the substrate (Simulation 5). The parameters for simulation were used as in Supplemental Fig. 1, and the initial sperm configuration, the views, and colors are given as in Supplemental Movie 3.

Supplemental Movie 6

The eggs were rendered buoyant by injection of paraffin oil into their perivitelline space. The paraffin oil-loaded eggs were floated to the surface of the medium. The spermatozoa could then exert their tail beating force from the lower side of the eggs. As predicted by the simulation, the floated eggs were shown to rotate in a clockwise direction.
